# Dr. Petermann's Dental Almanac for 1879

**Published:** 1879-05

**Authors:** 


### BIBLIOGRAPHICAL.

*Dr. Adolph Petermann's Dental Almanac*, 1879. Published by John Alt, in Frankfort on the Main. For sale by all Dental Depots. Price $1.00.

This Almanac contains the names arranged in alphabetical order, and the addresses of more than six hundred dentists practicing in Germany, Austria and Hungary, with two steel engravings of Drs. D. Fricke, Sen., and the late Dr. F. Turnovsky (See Editorial.)

